# Alternative dispute resolution: Mediation as a model

**DOI:** 10.12688/f1000research.152362.1

**Published:** 2024-07-09

**Authors:** Naser Sherman, Bashar Talal Momani

**Affiliations:** 1 Law, American University in the Emirates, Dubai, United Arab Emirates

**Keywords:** Mediation, Mediator, Civil and Commercial Disputes, Arab Legal systems

## Abstract

**Background:**

The topic of Alternative Dispute Resolution (ADR) in civil and commercial contexts presents a contemporary legal challenge aimed at fostering equitable solutions. Among ADR methods, mediation stands out for its ability to reduce time, costs, and litigation duration. This study explores the conceptual framework, essential conditions, and procedural aspects of mediation. It evaluates the sufficiency, regulation, and effectiveness of mediation principles in conflict resolution and risk mitigation.

**Methods:**

This study is conducted through a comprehensive review of current literature on Alternative Dispute Resolution methods, with a particular focus on mediation as an alternative to dispute settlement. The study utilizes qualitative analysis techniques to examine the effectiveness of mediation principles and their application in resolving civil and commercial disputes. Comparative analyses are also conducted to extract useful insights from various legal systems and authorities.

**Results:**

The study provides an analysis that illustrates the effectiveness of mediation in resolving disputes, emphasizing its potential benefits in terms of time and cost savings, as well as its srole in facilitating amicable resolutions. The results of this study shall contribute to the current body of knowledge on mediation and provide practical recommendations for its application in diverse legal contexts.

**Conclusion:**

In conclusion, the study proposes strategies to enhance mediation practices, promote a culture of its adoption, and integrate it more closely into the judicial system. Additionally, it anticipates the future effectiveness of mediation in jurisdictions lacking comprehensive legislation, drawing from successful Western experiences to guide potential developments in Arab legal frameworks.

## 1. Introduction

Mediation is a general idea that has existed in societies since its inception. However, it did not gain momentum as a legal idea except in contemporary civil and commercial laws. It appeared due to the slowness and complexity of the judiciary in judicial procedures as disputes may take a long time and more expenses, including experts, lawyers, and court fees inter alia. We may add to that the state of hatred between litigants when the court ruling is issued because the judgment in judicial disputes may not be satisfactory to both parties, but it is rather in favor of one over the other. This may be justified for logical and legal reasons. The judiciary seeks to reach the judicial truth which may be in agreement or disagreement with the realistic truth. Mediation was primarily introduced for the purpose of solving these problems. By virtue of this mediation, a third party takes over the study of the dispute and elaborates a solution and a settlement that satisfies the two parties to the dispute. It is an advanced method of resolving disputes between the parties by their agreement. The parties appoint a person called the mediator, whose task is to limit the dispute through the continuity of communication between the parties individually and jointly with the aim of bridging the gap between them so as to meet and agree eventually on a mutual compromise. Mediation then, is a voluntary process in nature, and the mediator may not make a decision that has become the basis of the dispute. Rather, her/his role is limited to trying to conciliate the opposing views of the parties and to proposing alternative solutions before them without imposing any of these on them.

The research problem revolves around the fact that most of the legislations have not codified legal mediation in an independent law that regulates it as an alternative to resolving disputes and that its provisions are distributed or spread in many laws. Therefore, this paper tries to answer the following questions:

• Can mediation resolve commercial and civil disputes?
• Is mediations considered an alternative to the judiciary in resolving these disputes?
• What are the requirements of a mediator?

## 2. Literature review

Mediation is one of the most preoccupying topics for many scholars and researchers given its vital role in resolving civil and commercial disputes. It is also efficient as it saves time ( Shi’an and Ahmed, 2017; Hamdan and Frederick, 2014), the topic has been the subject of numerous case studies ( Al-Naqash, 2009; Badawi, 2003), unlike ours, have addressed the procedural and confidential methods of resolving disputes ( Abdel Reza, 2015; Aqeelah, 2011) and emphasize its role as an alternative method ( Dheeb, 2009; Al-Qurtubi, 1985; Ben Belkacem, 2009; Odilqoriev, 2022; Fuad Abd Al-Baqi et al., n.d.; Al-Ahmad, 2008, n.d.; Al-Salibi, 2010, n.d.; Mubarak, 2002; Sadiq, 1982; Al-Nasser and Al-Ghanem, 2003; She’an and Ahmed, 2014, n.d.; Zaki, 1993; Al-Qudah, 2004; Mustafa, 1952; Sloma, 2023; Al-Khafaji and Al-Khafagi, 2021; Cachard, 2008; Jean Louis Lascoux, 2009).‏Valentyna Sloma’s study, entitled “Mediation as a Method of Resolving Civil Legal Disputes” ( Sloma, 2023), focuses on defining.

Valentyna Sloma’s study, entitled “Mediation as a Method of Resolving Civil Legal Disputes”, focuses on defining mediation and its characteristics as an extrajudicial, voluntary, and confidential method. The study highlights mediation’s effectiveness in property law disputes and emphasizes its role as an alternative method for resolving civil legal disputes.

Frank E.A. Sander’s “Alternative Methods of Dispute Resolution: An Overview” underscores the importance of continuous research and experimentation in developing a conceptual framework for alternative dispute resolution. It aims to raise awareness about the benefits of alternative methods of resolving disputes.

Faras Karim Shi’an and Hind Faiz Ahmed’s study, “Mediation in Electronic Disputes,” examines mediation in electronic disputes under Iraqi laws and international agreements. The study discusses the absence of mediation in Iraqi legislation and its effectiveness in settling disputes.

Mohamed Nabil Naqash’s study, “Banking and Insurance Mediation in Tunisian Law,” analyzes mediation and conciliation in banking operations in Tunisian law. It highlights the prevalence of mediation in commercial disputes and advocates its use before resorting to courts.

Mounir Mahmoud Badawy’s study, “Mediation and Third-party Role in Dispute Resolution,” explores the mediator’s role in conducting mediation procedures and facilitating settlement solutions. The study emphasizes mediation as a successful means for resolving disputes and conflicts.

Mohammed Ali Abdel Reza’s study, “Mediation in the Peaceful Resolution of Disputes in Iraqi Legislation: A Comparative Study,” investigates mediation as a means of amicable dispute resolution in Iraqi legislation. It addresses various issues related to mediation and emphasizes the mediator’s role in achieving speedy resolutions.

Nahla Yassin Hamdan and Frederick Pearson’s study examines disputes within companies and advocates extrajudicial settlement as the optimal approach. The study identifies governance issues leading to disputes and emphasizes the need for non-judicial resolution methods.

Forqan Ali Hussein Al-Khafaji and Hussein Al-Khafagi’s study addresses mediation as an alternative means of resolving disputes and the necessity of Iraqi legislation to regulate it. The study highlights the importance of legislative regulation of mediation and other alternative dispute resolution methods in Iraq.

What distinguishes our study from previous ones is its particular focus on the procedural aspects of the mediation process so as to achieve a resolution in civil and commercial disputes. While previous studies primarily highlighted the benefits of mediation and analyzed its role in resolving conflicts, our study goes beyond that to provide a comprehensive view of the mediation process, including its procedural aspects.

We shall provide detailed guidance on how to apply mediation as a method for resolving disputes, starting from the inception of the process to reaching a final settlement. We shall outline the necessary steps to initiate mediation, such as selecting the appropriate mediator, establishing confidentiality procedures, and collaborating with involved parties. Additionally, we shall analyze the factors that may impact the success of the mediation process and how to address them.

As such, our study stands out as a practical reference for researchers and stakeholders interested in the mediation process, offering specific and detailed guidance on organizing and executing mediation in an effective and fruitful manner.

This study is in line with the Mediation and Conciliation in Civil and Commercial Disputes Law, enacted by the United Arab Emirates in 2023. This law aims to enhance justice and facilitate the resolution of disputes through non-judicial means. It comes within the UAE’s continuous efforts to foster a favorable business environment and promote a culture of settlement and mediation.

This law represents a significant step towards enhancing mediation as a primary method for resolving disputes, providing the necessary legal framework to regulate and facilitate mediation and conciliation processes. The law includes provisions that specify the conditions and procedures necessary for conducting mediation, such as appointing qualified mediators, ensuring confidentiality of information, and defining procedures for reaching final settlements.

Furthermore, the law provides legal protection for parties participating in mediation processes, encouraging active engagement and fostering confidence in the outcomes. Additionally, the law works to promote a culture of conciliation and peaceful dispute resolution, in order to contribute to improving the business climate and attracting investors to the country.

Overall, the Mediation and Conciliation in Civil and Commercial Disputes Law for the year 2023 in the United Arab Emirates represents a significant step towards enhancing justice and stability in the legal environment and achieving fair resolution of disputes.

## 3. Methods

The methodology developed in this study adopts the descriptive analytical approach to the different principles of arbitration. The research will seek to provide an in-depth analysis to critically assess the importance of mediation as one of the effective and equitable methods of arbitration. This study draws upon basic resources, including but not limited to literary publications, scholarly periodicals, and online articles.

Through the methodology adopted in this study, which relies on the analytical approach, researchers from various countries can directly benefit from this research. They can utilize the theoretical and methodological framework elucidated here to conduct similar studies focusing on the importance and effectiveness of mediation as an equitable and effective method in arbitration. Furthermore, researchers can draw from the primary sources used, including literary publications, scholarly journals, and online articles, to support their analyses and academic discussions.

In this way, your study becomes a valuable contribution that the global academic community can benefit from in deepening their understanding of the importance of mediation as an effective tool in arbitration. It also encourages them to conduct further research to advance this field in their own countries and local communities.

## 4. Concept and origins

Mediation is based on the provision of some kind of dialogue to the conflicting parties to meet and converge views through an impartial person who assumes this task in order to reach an amicable solution that satisfies all the conflicting parties ( Sloma, 2023).

Article 8 of UAE Law No. 40 of 2023 concerning Mediation and Reconciliation in Civil and Commercial Disputes states: Article (8):

Mediation is permissible for all civil and commercial disputes that are amenable to resolution, provided it does not contravene applicable laws, public order, or morals within the state, taking into account the provisions of Article (28) of this law, and without affecting the provisions of local laws governing mediation. Mediation may encompass the entirety of a dispute or a portion thereof. The provisions of mediation outlined in this chapter shall apply under the following circumstances:

a. if mediation procedures are conducted within the state;
b. if mediation involves an international commercial dispute outside the state, and the parties agree to abide by the provisions of this law;

In the 1960s, the American Civil Court introduced the “American Rule”. This rule means that the parties to the dispute are obliged to pay lawyers, regardless of the success or loss of their case ( Odilqoriev, X.T., 2022). In doing so, the application of this rule has resulted in substantial costs and exorbitant amounts for the parties. The United States is one of the countries that relies on mediation in the resolving of the civil disputes.

In USA, mediation plays a key role in the resolution of extrajudicial disputes due to the complexities of the judicial system, and the high costs of litigation, especially lawyers’ fees because lawyers receive their fees in a manner consistent with their working hours to reach the judicial judgment in the dispute. Mediation in American laws initially emerged in relation to the resolution of labor disputes, especially with the enactment of the Addiction Act in 1898 ( Sloma, 2023),while, after the enactment of the Railway Labor Law in 1926, the commissions of mediation and reconciliation were formed to deal with labor issues. Mediation was not only limited to labor disputes. It was used as an effective means of resolving family disputes in the United States, where the Judges’ Association and Family Courts were established, with the aim of promoting reconciliation in family cases, as an alternative to litigation. These institutions were subsequently established across various regions of the USA, including the Family Mediation Association and the Family Mediation Academy ( JeanRobert, 1993; Elias Nassif, 2012; Musaada, Ayman, 2004). After that, mediation has become a mandatory procedure in USA family affairs, particularly for the issues related to marriage and divorce ( Agnéstavel & Lascoux, Jean Louis, 2009).

In the Latin legal system, mediation has been used since the end of the nineteenth century. Napoleon Bonaparte enacted a mediation law in 1803. The American mediation model prevailed in Germany, while it was less applied in France. There, the mediation system, from the outset, has been directly integrated within the justice courts ( Odilqoriev, X.T., 2022). Article (48) of the French Code of Civil Procedure of 1806 stipulates that the initiation of any lawsuit regarding a judicial dispute is inadmissible unless it is preceded by an attempt at preliminary reconciliation before the Magistrate’s Court. By introducing this article, it appears that the French legislator has given the litigants an opportunity to seek alternative means of resolving their disputes before levelling it before the court.

Mediation, from a formal standpoint, is an alternative litigation mechanism that aims to resolve the dispute, through the intervention of a neutral person, called a mediator, who works to help the parties to the conflict negotiate, in order to reach a settlement to resolve the dispute. The third paragraph of Article 1 of the UNCITRAL Model Law of the Year (2002) for international commercial conciliation defines mediation as “any process, whether referred to as conciliation or mediation, or by another term of similar meaning, in which the two parties request another person or persons (conciliator or conciliators) to assist them in their endeavors to reach an amicable settlement of their dispute arising out of a contractual or other legal relationship.”

In light of the foregoing, mediation can be defined as a voluntary process based on the will of the parties to the conflict to resort to it, and in which they work with a third person, called a mediator, who enjoys the capacity of integrity and impartiality and who can find a mutually acceptable solution that ends the dispute ( Mustil arbitration, 1980).

Mediation has become a prominent tool in legal and economic thought. At the global level, it is an urgent matter to meet the requirements of modern business due to the rapid and continuous expansion and development of trade patterns ( Al-Khafaji & Al-Khafagi, 2021) on the one hand. On the other hand, mediation has characteristics that distinguish it from traditional means of resolving disputes. These advantages have become acceptable for resolving disputes, reducing the burden on the judiciary and speeding up disputes solving with flexibility.

There is no doubt that slow justice is a denial of justice. So, we argue that the mediation process largely serves this purpose, namely, the rapid pace of settling disputes. This is shown by the legislator’s determination of the period in which mediation is accomplished. The Jordanian legislator goes further, as it sets the period for which the parties restrict the provision of the mediator with documents related to the dispute starting from the date of referring the dispute to mediation. The purpose is to ensure the resolution of disputes at a rapid pace as mediation avoids complicated formalities. Several requirements must be followed on pain of nullity, imposing restrictions on the parties involved in the litigation. In mediation, there is no procedure resulting in nullity, so on the contrary, mediation aims to follow any procedure that could lead to a satisfactory solution to the parties to the dispute. Moreover, resorting to mediation is not mandatory. It is the right of any of the parties to refuse mediation and resort to the judiciary to proceed with the case in accordance with what is legally prescribed for it. The parties to the conflict derive this right from the constitution.

However, it must be noted that mediation differs from arbitration, as this is a means of settling disputes in which the arbitrator’s ruling on the subject matter of the dispute replaces the judicial ruling. Accordingly, there are those who see that the position of the arbitrator is identical to that of the judge, even if their sources differ ( David (René), 1982; Abdul Al-Qadir, Nariman, 1996). The parties agree to select the members of the arbitration tribunal to decide on the issue in dispute. The arbitrator shall have the role of a judge in resolving the dispute subject to the arbitration agreement, by an arbitration award that is binding on them, and stems from the will of the arbitrators and not the will of the parties to the dispute. Arbitration is binding. As for mediation, it is not. Also, the judgment issued by the arbitration tribunal, or the arbitrator shall be considered an executive document whenever the order for its implementation is issued by the public judiciary in the state, and it is challenged by the legally prescribed appeal methods. Just as mediation differs from a legal point of view from reconciliation, as this latter according to the provisions of the civil law, is a contract by which the two parties actually resolve a conflict that has arisen between them, or prevent a potential conflict. This results from each party’s voluntary relinquishment of some of its demands ( Camara Fatou kiné, 2009). As for mediation, it is the endeavors undertaken by the two parties with the mediator to help the conflicting parties, to reach an agreement on a solution that ends the dispute amicably, instead of initiating legal procedures. So, mediation is a means; as for reconciliation, it is not a means, but rather an end in nature. And this is done by reaching an agreement on reconciliation ( Kanakria, Walid, 2020).

### 4.1 The qualities of the mediators

From a legal point of view, a mediator is the person who undertakes the implementation of the mediation process, s/he must have a set of characteristics, and s/he must seek to bring the disputants to a settlement that they are satisfied with ( Lord Mustill & Stewart C Body, 2001). In fact, Article 6 of UAE Law No. 40 of 2023 concerning Mediation and Reconciliation prohibits both the mediator and the reconciler from acting as an arbitrator or expert in the dispute, or accepting delegation in a dispute against any of the parties regarding the subject of the mediation or reconciliation, or any related matter, even after the conclusion of the mediation or reconciliation proceedings, unless the parties agree otherwise regarding the mediation. Also, it prohibits giving testimony against any party to the dispute concerning the subject of the mediation or reconciliation, or any related matter, even after the conclusion of the mediation or reconciliation proceedings, unless authorized by the concerned party or agreed upon by the parties to the contrary, except in cases involving a crime. It prohibits as well acting as a mediator or reconciler in a dispute where one of the parties is a spouse or relative up to the fourth degree by blood or marriage.

The qualities that must be present in the mediator can be limited to the following:

• **Legal capacity**

The mediator must be fully qualified, and the eligibility condition is a fundamental issue required in all jobs and tasks. On the obstacles and difficulties encountered, the mediation process requires, during all its stages, a mediator with sophistication, acumen, and personal skills that qualify him to manage negotiations ( Jalloul, Dalila, 2012). Article (31) of UAE Law No. 40 of 2023 concerning Mediation and Reconciliation stipulates the conditions for occupying the position of mediator. It states that: the conditions for appointing reconcilers and mediators and their qualification shall be determined by a decision of the council or the head of the local judicial authority, as deemed appropriate. It shall include the following conditions: not to have lost eligibility or been convicted of a crime involving honor or trustworthiness, even if her/his status has been restored. Another condition is to be known for honesty, neutrality, and experience. A further one is to successfully complete specific courses and examinations, determined by a decision of the council or the head of the local judicial authority, as deemed appropriate.

• **Neutrality and impartiality**

Neutrality means liberating the mind from all fanaticism and preparing it with all that is acceptable to the law, and the impression of justice. Neutrality represents the first characteristic of the mediation process, as it takes multiple implications, and is defined in a variety of ways. One side should not be favored over the other ( David (René), 1982).

Experience and competence have special importance in settling disputes through mediation because an experienced and competent mediator gains more confidence in her/himself first and in the mediation process second. Experience and competence provide the mediator with the trust of the parties in her/his person and her/his ability to settle the dispute and ultimately they get satisfaction with her/his judgment and agree to implement it. The mediator must also be legally qualified in both academic and practical terms, in a manner that enables her/him to conduct the mediation process and achieve the desired goal in it with high efficiency and capacity. (Ibid, at 266-67) This can only be achieved through training and legal preparation in accordance with intensive theoretical and practical programs in the field of mediation and dispute settlement, as well as the knowledge of legislation which should foster the ability of the mediator in settling disputes ( Peter S. Caldwell, 1992).

### 4.2. Legal mediation procedures

Mediation is inherently a voluntary process in which the mediator is not permitted to make decisions on the core issues of the dispute ( Cachard, 2008). Rather, her/his role is limited to trying to bring the two parties’ points of view closer without being bound by the procedures stipulated in the Civil Procedures Law. However, this does not mean that there are no restrictions or procedural rules that the mediator adheres to. The mediator must carry out her/his work according to specific principles that help her/him deliver the litigants to a solution satisfactory to both parties. Mediation is based on supportive negotiation that leads to resolving the conflict in the shortest time, with an agreement the strength of which lies in the self-determination, as the mediator seeks to bridge the views between the opponents who disclose to the mediator their basic interests, and their anxiety factors, in order to enable her/him to find solutions that are satisfactory to all parties ( Aflouk, Muhammad Ali Abd Al-Redha & Al-Zubaidi, Yasser Ataiwi, 2015). The procedures can be divided into two types: rules related to starting and conducting mediation and others related to its termination.

Article (18) of the UAE Mediation and Reconciliation Law for the year 2023 outlines the mediation procedures as follows:

The mediator must inform the disputing parties of the mediation sessions and officially announce their date, time, and location through any of the legally prescribed means of communication, including electronic methods.

The disputing parties are required to attend the mediation sessions personally or through a legal representative, with proper authorization. If one of the parties is a legal entity, their legal representative or authorized agent must attend. Parties may also engage advisors to accompany them during the sessions. The mediator has the discretion to determine the number of individuals accompanying each party based on what is deemed suitable for facilitating the settlement process considering the circumstances and nature of the dispute. Attendance of non-parties in the dispute requires the consent of all parties involved.

Each party must submit to the mediator, before the first scheduled session, a concise memorandum summarizing their claims or defenses, along with the documents and evidence supporting them. These memoranda and documents are not to be exchanged between the parties.

Article (18) of the UAE Mediation and Reconciliation Law for the year 2023.

• **Procedures for initiating & conducting mediation**

The first session is an introduction session in which the mediator introduces her/himself and the litigantsto introduce themselves. S/he explains to them her/his role as a mediator, and affirms her/his neutrality and the confidentiality of procedures. The trust of the parties in the mediator is more important than their trust in each other ( Sander, 1985).

The mediator must keep the secrets that s/he finds, because these secrets have reached her/his own person by virtue of her/his work as a mediator. If it were not the case, s/he would not obtain them. So, the secrets that s/he must keep include the information and documents that s/he has access to for her/his capacity as a mediator ( Al-Tahiwi, Mahmoud Al-Sayed 2006). As a personal trait, the more competent and capable the mediator is, the more likely s/he can reach an agreement that satisfies the conflicting parties ( Okechukwu Dominic, 2012). There are several methods that the mediator follows to reach this agreement, the most important of which are:

• **Clarifying ideas and providing the necessary facilities:**

This method is based on clarifying to the parties’ ideas and demands, and placing them within their correct frameworks. The mediator shall make the necessary facilities and provide assistance to the parties in communication and negotiation ( Odega, Bensalem, 2009) between them within the framework of a strategy. This strategy is designed to take the parties’ hand to identify the topics and issues related to the conflict **(Ibid 83-89)** and a correct understanding of their needs and their true interests.

• **Evaluating facts and expressing an opinion:**

This method is represented by the mediator meeting with each of the parties to the dispute separately to assess her/his legal status and express her/his expected opinion regarding her/his case, by reviewing the legal texts and judicial jurisprudence in this regard. Here lies the importance of the mediator having scientific and practical experience that enables her/him to evaluate issues in a convincing manner that maintains confidence and impartiality. This requires, in addition to scientific and practical experience, familiarity with the training skills and technical methods used, in dealing with parties (Civil Litigation & Alternative Dispute Resolution, without date).

The mediator must have the moral characteristics required of a respectable person in order not to be subjected to question or suspicion, and the parties would not question whether they are in front of a mediator or an arbitrator. Only then, they can have the confidence that drives them to put all their affairs in the hands of this person, whom they know, trust and believe in her/his ability to provide them with assistance to get out of the impasse ( Al-Ahmad, at 118, n.d.). In addition to that, the mediator’s status and prestige binds the parties morally and socially to accept the solution that s/he offers them ( Sloma, 2023).

The role of the mediator is to correct the situation by asking questions about the claims and the answers that s/he has received. This may lead her/him to obtain facts that the parties neglected to mention which may help resolve the dispute ( Al-Khafaji & Al-Khafaji, 2021), and which may not occur otherwise. This is done by facilitating the means and methods of discussion between the parties to the conflict by converging viewpoints and opinions between them. It also happens by strengthening the parties’ sense of legal responsibility towards the issue and enhancing the importance of resolving the disputed issue via a set of legal alternatives, suitable for resolving the dispute. It is also crucial to preserve the independence of each of the parties to the conflict.

If the mediator reaches a settlement of the dispute, s/he submits a report to the case management judge or the magistrate judge and attaches the settlement agreement signed by the parties to the dispute to ratify it to be considered as a final judgment that is not subject to any method of appeal ( Badawi, Mounir Mahmoud, 2003). If the judge approves an agreement, mediation then comes to the implementation phase. The implementation of this agreement is voluntary in principle, because the parties have agreed on it ( Henry, M., 1993). Thus, they are considered to be more able to understand and abide by it.

Many of the laws that regulate mediation set a specific period for the mediator to reach a solution to the dispute, including what was stipulated in the Jordanian Mediation Law No. 37 of 2003. Provided that the mediation sessions take place in the presence of the litigant parties and their legal representatives, the mediator shall, within a period not exceeding three months from the date of referring the dispute to her/him, do the following: set a session, inform the parties to the dispute and their agents, deliberate according to the rules, meet with the parties to the dispute and their representatives, negotiate with them the subject of the dispute and take whatever s/he deems fit for bridging views for the purpose of reaching an amicable solution to the conflict. The Tunisian legislator also stipulates that the mediator in the field of banking must undertake the complaints submitted to her/him within a maximum deadline of eight days from the date of receiving the mediation request and proposes appropriate compromise solutions within a maximum deadline of two months from the date of the undertaking ( Kiné, Camara 2004).

• **Termination of the Mediation**

Mediation ends either by agreeing to resolve the dispute, or by not agreeing to resolve it. If the parties agree to end the dispute, this is considered a success for the mediator in her/his work. The mediator subsequently submits a report called the mediation agreement signed by the mediator and the parties to the dispute. Yet, this agreement may be wholly conducive to a complete solution to the dispute ( Cachard, 2008). It may be partial leading to the resolution of some issues, and others remain without agreement. Also, if the agreement is complete, the report is referred, after it is signed to the court for approval by the judge. If it is partial, it is written in the minutes that have been agreed upon and signed by the mediator and the parties. The file is referred to the judge, and the parties are bound by the signed agreement. The parts that have not been agreed upon are referred by the judge to the competent court for a final judgment ( Al-Salibi, Bashir, at 60, n.d.).

When the judge ratifies the mediation agreement, s/he does not ratify it as carrying out her/his function as the judicial authority ( Moneim, 1999). Rather, s/he ratifies it as the custodian of the state authority. Mediation ends in accordance with the provisions of the UNCITRAL Model Law on International Commercial Conciliation of 2002 in one of the following cases:

The first case: the mediator fails to reach an amicable settlement of the dispute between the litigants. In this case, there are numerous reasons, any of which may lead to the termination of the mediation process. Examples of these reasons are stated in Article (11) of the UNCITRAL Model Law on Commercial Conciliation. They are as follows:

1- After consulting with the parties to the conflict, a declaration is issued stating that there is no justification for undertaking further efforts at reconciliation on the date of the declaration.
2- A party to the dispute issues a declaration addressed to the other party, or other parties, and to the conciliator in the event that s/he is appointed, indicating that the conciliation procedures have ended on the date of the announcement.

The second case: when the mediator reaches an amicable settlement of the dispute between the litigants, and the settlement agreement is approved by the parties to the dispute, the mediation process ends at the moment of ratification of the settlement agreement.

The third case: the expiration of the period specified for the settlement of the dispute, as stipulated in the first paragraph of Article Seven of the Jordanian Mediation Law, which obliges the mediator to complete the mediation work within a period not exceeding three months from the date of referring the dispute to her/him.

The fourth case: the parties to the dispute have not attended the mediation sessions without a legitimate excuse, as stipulated in Article (5) Paragraph (B) of the Jordanian Mediation Law.

## 4. Mediation discrimination from arbitration

Mediation is an analytical and optional process where disputing parties choose a mediator to assist in resolving conflicts. The mediator facilitates communication and information exchange between the parties, offering guidance and support to reach mutually satisfactory solutions ( Jean Louis Lascoux, 2009). It is noteworthy that the mediator’s role is limited to providing a safe and neutral environment for parties to discuss the issue and reach a settlement without imposing decisions on them. Additionally, the mediator must be well-trained in techniques for successful conflict resolution and creative solutions ( Sloma, 2023).

The forms of mediation vary according to the nature and circumstances of the dispute. This includes simple mediation, which involves informal sessions with the mediator to facilitate dialogue and reach a settlement. There’s also formal mediation, which includes court procedures and the appointment of a panel chaired by the mediator to resolve disputes. There is consultation mediation as well which involves parties seeking advice from a lawyer or expert in the field before resorting to mediation to resolve the dispute ( Sloma, 2023).

One of the distinctive aspects of mediation is its flexibility in the dialogue and exchange of ideas, striving to ensure that each party is heard and their needs and interests are fairly addressed. It also works to promote understanding and build trust between the parties, making the solutions reached more sustainable and acceptable to all.

In contrast, arbitration is characterized by its formal and binding nature, where arbitrators decide disputes based on evidence presented and applicable laws, without the need for parties’ consent to the final decision. Arbitration is characterized by confidentiality and efficiency in issuing decisions, making it a common choice for disputes requiring a quick and binding resolution ( Al-Khafaji & Al-Khafagi, 2021).

In conclusion, while mediation seeks to achieve balance and cooperation between the parties, arbitration aims to issue final decisions that effectively and bindingly resolve disputes.

## 4. Conclusion

This research has made it clear that mediation and other alternative methods have become acceptable to litigants and appropriate for many disputes especially in civil and commercial disputes. No one can overlook their importance in international disputes, given the requirements of these types of disputes including flexibility and speed. They are also important in disputes arising from the use of modern means of communication, such as the internet, ecommerce, and disputes related to intellectual property, among others. The right of mediation is considered as an alternative to the judiciary to resolve these disputes. It should not be granted to every person or institution, but rather a mediator must have the qualifications to carry out the mediation process and the ability to provide solutions to the disputants without relying on legal texts, provided that they do not violate public order and public morals.

Therefore, we believe that it has become necessary to work on spreading and consolidating the culture of resorting to mediation. It has accordingly a positive role in reducing the burden on the judiciary. Thus, specialized courses and seminars should be held to qualify people who are able to conduct mediation. We also believe that the judicial mediation system must be activated within the judicial authority because the survival of mediation outside Legislation framework weakens its effectiveness in settling civil and commercial disputes. However, there rise the necessity to issue legislations regulating the mediation process as an alternative solution to settling civil and commercial disputes. It is also primordial to work to establish an independent special body or bodies that have special provisions and procedures that differ from what the judicial authority is subject to in supervising and controlling mediation between disputants.

## Data Availability

All data underlying the results are available as part of the article and no additional source data are required.
