# Supplementary material for: Alternative dispute resolution: Mediation as a model
Source: F1000Res. 2025 Jan 14;13:778. Originally published 2024 Jul 9. [Version 2] doi: 10.12688/f1000research.152362.2 (PMC11806251; doi:10.12688/f1000research.152362.2)
Supplement: Peer review history

# Reviewer report: Reviewer response for version 2

**DOI:** 10.5256/f1000research.175829.r359023

**Published:** 2025-02-08

**Authors:** Enas Mohammed Al-Qodsi

**Affiliations:** 1 United Arab Emirates University, Emirate of Abu Dhabi,, United Arab Emirates

I would like to thank the author for the revisions made to the manuscript, which have enhanced its clarity and precision. The study provides a valuable contribution to the discussion on mediation as an Alternative Dispute Resolution method, highlighting its effectiveness in resolving civil and commercial disputes. The comparative analysis further enriches the discussion and adds an important dimension to the legal debate.

 I have no further comments and appreciate the effort invested in preparing this research.

Is the work clearly and accurately presented and does it cite the current literature?

Yes

If applicable, is the statistical analysis and its interpretation appropriate?

I cannot comment. A qualified statistician is required.

Are all the source data underlying the results available to ensure full reproducibility?

Yes

Is the study design appropriate and is the work technically sound?

Yes

Are the conclusions drawn adequately supported by the results?

Partly

Are sufficient details of methods and analysis provided to allow replication by others?

Yes

Reviewer Expertise:

Private Law – Civil Liability Law – Artificial Intelligence

I confirm that I have read this submission and believe that I have an appropriate level of expertise to confirm that it is of an acceptable scientific standard.

# Reviewer report: Reviewer response for version 2

**DOI:** 10.5256/f1000research.175829.r359022

**Published:** 2025-02-07

**Authors:** Eka An Aqimuddin

**Affiliations:** 1 Universitas Islam Bandung, Bandung, West Java, Indonesia

Authors already revised the article adequately to meet journal aims.

Is the work clearly and accurately presented and does it cite the current literature?

Partly

If applicable, is the statistical analysis and its interpretation appropriate?

Partly

Are all the source data underlying the results available to ensure full reproducibility?

Partly

Is the study design appropriate and is the work technically sound?

Yes

Are the conclusions drawn adequately supported by the results?

Yes

Are sufficient details of methods and analysis provided to allow replication by others?

Partly

Reviewer Expertise:

International law, International Settlement of Dispute

I confirm that I have read this submission and believe that I have an appropriate level of expertise to confirm that it is of an acceptable scientific standard.

# Reviewer report: Reviewer response for version 1

**DOI:** 10.5256/f1000research.167111.r307497

**Published:** 2024-10-10

**Authors:** Eka An Aqimuddin

**Affiliations:** 1 Universitas Islam Bandung, Bandung, West Java, Indonesia

This article only represents the mediation in United Arab Emirates. If the author would like a broader audience, it should be compared to the mediation in another country.

 Perhaps the author can make a comparison with ADR in Europe or another continent.

 This article also did not give a new insight of mediation. The conclusion of mediation as ADR is common in another part of the world. As a reader, I did not get fresh knowledge from this article.

 In sum, there is no problem question you would like resolved.

 In my review, the article has no research questions or legal issues. Consequently, it does not enhance the discourse in public, especially the academic community.

 The discourse in the article is very confined. It requires expansion through comparisons with practices in other nations. By employing this approach will elucidate differences and similarities, providing the reader with a novel viewpoint.

Is the work clearly and accurately presented and does it cite the current literature?

Partly

If applicable, is the statistical analysis and its interpretation appropriate?

Partly

Are all the source data underlying the results available to ensure full reproducibility?

Partly

Is the study design appropriate and is the work technically sound?

Yes

Are the conclusions drawn adequately supported by the results?

Yes

Are sufficient details of methods and analysis provided to allow replication by others?

Partly

Reviewer Expertise:

International law, International Settlement of Dispute

I confirm that I have read this submission and believe that I have an appropriate level of expertise to state that I do not consider it to be of an acceptable scientific standard, for reasons outlined above.

# Reviewer report: Reviewer response for version 1

**DOI:** 10.5256/f1000research.167111.r311826

**Published:** 2024-08-22

**Authors:** Enas Mohammed Al-Qodsi

**Affiliations:** 1 United Arab Emirates University, Emirate of Abu Dhabi,, United Arab Emirates

The research paper focuses on Alternative Dispute Resolution (ADR), with a specific emphasis on mediation as a model. It explores the benefits of mediation compared to traditional litigation, highlighting advantages such as cost-effectiveness, time efficiency, and the preservation of relationships between disputing parties. The paper also discusses the principles, processes, and challenges of mediation, emphasizing its flexibility, voluntary nature, and confidentiality, which allow it to adapt to the needs of the parties involved.

 Evaluation of Key Points:

 1. Clarity of Concepts:

 Strengths: The research clearly defines and explains the concept of mediation and its role within the broader context of ADR.

 Suggestions: The clarity of the concepts can be further enhanced by providing more practical examples that distinguish mediation from other forms of ADR, such as arbitration.

 2. Research Methodology:

 Strengths: The paper utilizes a critical and comparative analysis to evaluate mediation against other dispute resolution methods.

 Suggestions: The methodology could be detailed further, particularly regarding how data was collected and analyzed, and how case studies (if any) were selected. A clear explanation of the research process will enhance the reliability of the findings.

 3. Case Studies or Practical Examples:

 Strengths: The paper effectively discusses the theoretical benefits of mediation.

 Suggestions: It would be beneficial to include case studies or practical examples that support the theoretical claims. This addition would strengthen the paper by providing concrete evidence of mediations' effectiveness.

 4. Discussion of Challenges:

 Strengths: The research strongly focuses on the advantages of mediation.

 Suggestions: A more balanced discussion is recommended, including potential challenges associated with mediation, such as power imbalances between parties and situations where mediation may not be appropriate. Addressing how these challenges can be mitigated would provide a more comprehensive analysis.

 Key Points to be Addressed:

 To ensure the research is scientifically sound, the following aspects should be clearly addressed:

 Strengthening Theoretical Foundation: The paper should establish a solid theoretical foundation by clearly defining key concepts and explaining their relevance to the topic of mediation.

 Enhancing Methodological Rigor: The authors should ensure that the methodology is transparent, systematic, and reproducible. This includes explaining how data was collected and analyzed and acknowledging any limitations or biases.

 Providing Empirical Support: The research should be backed by empirical evidence, such as case studies or data analysis, to substantiate theoretical claims.

 Balanced Perspective: The paper should offer a balanced discussion that not only highlights the benefits of mediation but also addresses potential challenges and how they can be overcome.

Is the work clearly and accurately presented and does it cite the current literature?

Yes

If applicable, is the statistical analysis and its interpretation appropriate?

I cannot comment. A qualified statistician is required.

Are all the source data underlying the results available to ensure full reproducibility?

Yes

Is the study design appropriate and is the work technically sound?

Yes

Are the conclusions drawn adequately supported by the results?

Partly

Are sufficient details of methods and analysis provided to allow replication by others?

Yes

Reviewer Expertise:

Civil LawCivil LiabilityArtificial IntelligenceContractsSustainable Development

I confirm that I have read this submission and believe that I have an appropriate level of expertise to confirm that it is of an acceptable scientific standard.

## References

[1] : A legal framework for online commercial arbitration in UAE: new fabric but old style!. *Information & Communications Technology Law* .2017;26(3) : 10.1080/13600834.2017.1374055 272-292 10.1080/13600834.2017.1374055

# Response

**Published:** 2024-08-22

**Authors:** Naser Sherman

**Affiliations:** Law, American University in the Emirates, united arab emirates - dubai, Dubai Academic City, United Arab Emirates

Dear Reviewer,

 Thank you very much for your thorough and detailed review of the research. Your comments are extremely valuable and will undoubtedly contribute to improving the quality of the work. I would like to address each of the points you raised:

 1. Clarity of Concepts:

    - Response: I appreciate your recognition of the clarity in defining mediation and its role within Alternative Dispute Resolution (ADR). It is important to clarify that mediation is a method of amicable dispute resolution, and distinguishing it from other ADR methods, such as arbitration, is crucial. This will certainly help in enhancing the clarity of the concepts.

 2. Research Methodology:

    - Response: Thank you for acknowledging the use of critical and comparative analysis. I agree with you, and I have provided a brief explanation of the research methodology. The criteria for selecting case studies focused mainly on the most recent studies.

 3. Case Studies or Practical Examples**:

    - Response: Case studies or practical examples have been briefly included in the paper to support the theoretical benefits of mediation.

 4. Discussion of Challenges:

    - Response: Your recommendation to provide a more balanced discussion that includes the potential challenges of mediation is appreciated. Regarding overcoming these challenges, they can be addressed by enacting modern legislation that accurately regulates mediation. Once implemented, these laws will reveal challenges that can then be addressed with appropriate proposals.

 5. General Feedback:

    - Response: The theoretical foundation has been strengthened, methodological rigor ensured, and empirical support along with practical examples provided.

 Regarding the statistical analysis, since this paper primarily focuses on qualitative analysis, there was no need for detailed statistical interpretation.

 Thank you once again for your constructive feedback. I am committed to addressing these points and improving the quality of the paper.
